# Beta-band oscillations in the supplementary motor cortex are modulated by levodopa and associated with functional activity in the basal ganglia

**DOI:** 10.1016/j.nicl.2018.05.021

**Published:** 2018-05-18

**Authors:** Jae Woo Chung, Roxana G. Burciu, Edward Ofori, Stephen A. Coombes, Evangelos A. Christou, Michael S. Okun, Christopher W. Hess, David E. Vaillancourt

**Affiliations:** a Department of Applied Physiology and Kinesiology, University of Florida, Gainesville, FL, USA; b Department of Kinesiology and Applied Physiology, University of Delaware, Newark, DE, USA; c College of Health Solutions, Arizona State University, Phoenix, AZ, USA; d Department of Physical Therapy, University of Florida, Gainesville, FL, USA; e Department of Neurology and Center for Movement Disorders and Neurorestoration, University of Florida, Gainesville, FL, USA; f Department of Biomedical Engineering, University of Florida, Gainesville, FL, USA

**Keywords:** BOLD, blood oxygen level dependent, DBS, deep brain stimulation, ECoG, electrocorticography, EEG, electroencephalography, EMG, electromyography, ERSP, event-related power spectral perturbation, FDR, false discovery rate, fMRI, functional magnetic resonance imaging, HC, healthy control, ICA, independent component analysis, iEMG, integrated electromyography, LFP, local field potential, M1, primary motor cortex, MDS-UPDRS, Movement Disorder Society Unified Parkinson's Disease Rating Scale, MEG, magnetoencephalography, MoCA, Montreal Cognitive Assessment, MPA, measure projection analysis, MVC, maximum voluntary contraction, PET, positron emission tomography, PD, Parkinson's disease, PD-OFF, off medication (levodopa) day, PD-ON, on medication (levodopa) day, rCBF, regional cerebral blood flow, ROI, regions of interest, S1, primary somatosensory cortex, SMA, supplementary motor area, SNc, substantia nigra pars compacta, STN, subthalamic nucleus, Parkinson's disease, Ballistic movements, Levodopa, EEG, Supplementary motor area, fMRI

## Abstract

We investigated the effect of acute levodopa administration on movement-related cortical oscillations and movement velocity in Parkinson's disease (PD). Patients with PD on and off medication and age- and sex-matched healthy controls performed a ballistic upper limb flexion movement as fast and accurately as possible while cortical oscillations were recorded with high-density electroencephalography. Patients off medication were also studied using task-based functional magnetic resonance imaging (fMRI) using a force control paradigm. Percent signal change of functional activity during the force control task was calculated for the putamen and subthalamic nucleus (STN) contralateral to the hand tested. We found that patients with PD off medication had an exaggerated movement-related beta-band (13–30 Hz) desynchronization in the supplementary motor area (SMA) compared to controls. In PD, spectral power in the beta-band was correlated with movement velocity. Following an acute dose of levodopa, we observed that the beta-band desynchronization in the SMA was reduced in PD, and was associated with increased movement velocity and increased voltage of agonist muscle activity. Further, using fMRI we found that the functional activity in the putamen and STN in the off medication state, was related to how responsive that cortical oscillations in the SMA of PD were to levodopa. Collectively, these findings provide the first direct evaluation of how movement-related cortical oscillations relate to movement velocity during the ballistic phase of movement in PD and demonstrate that functional brain activity in the basal ganglia pathways relate to the effects of dopaminergic medication on cortical neuronal oscillations during movement.

## Highlights

• Acute levodopa decreased beta-band desynchronization in the SMA, while improving movement velocity and muscle activity.
• Beta-band cortical activity during movement is positively correlated with upper limb movement velocity.
• fMRI in basal ganglia predicted the response of beta-band cortical activity to levodopa.

## 1 Introduction

Since the time of Woodworth's seminal studies in motor behavior, we have known that the two key phases in the performance of a voluntary movement are the ballistic phase and the corrective phase (Woodworth, 1899). The initial ballistic phase consists of a fast pre-programmed movement that occurs without feedback (referred to as an “open-loop” movement) while the subsequent corrective phase utilizes proprioceptive and visual feedback to increase movement accuracy. Later studies established the characteristic triphasic pattern of agonist and antagonist electromyography (EMG) activity during ballistic movements, in which an initial agonist EMG burst accelerates the limb toward the target and is followed by an antagonist EMG burst that decelerates the limb as it approaches the target and a final stabilizing burst of agonist EMG activity (Hallett et al., 1975). The acceleration and magnitude of the initial EMG burst has been shown to scale with movement velocity (Gottlieb et al., 1989).

In Parkinson's disease (PD) movements are performed at a reduced velocity, referred to as bradykinesia. The onset of bradykinesia is associated with a selective loss of dopaminergic cells in the substantia nigra pars compacta (SNc) (Fearnley and Lees, 1991; Stocchi and Olanow, 2003). In patients with PD who perform a ballistic movement, the typical triphasic pattern is altered, with fractionated agonist EMG bursts that are marked by reduced agonist burst duration and magnitude, and an increase in the number of triphasic burst cycles. Dopaminergic medication and deep brain stimulation (DBS) of the subthalamic nucleus (STN) have been shown to increase the voltage of the first agonist burst and reduce fractionation of agonist activity (Hallett et al., 1975; Pfann et al., 2001; Vaillancourt et al., 2004). While there has been considerable focus on EMG activity during a ballistic movement, we know far less about how brain activity changes during a ballistic movement in patients with PD.

When using electrophysiological recording methods in the basal ganglia and motor cortex, a consistent observation is that beta-band (13–30 Hz) oscillations decrease in power during a movement (Brown and Marsden, 1999; Kühn et al., 2004; Heinrichs-Graham et al., 2017). In healthy controls, beta-band cortical oscillatory activity is known to be associated with motor control and learning (Pfurtscheller and Lopes da Silva, 1999; Engel and Fries, 2010; Kilavik et al., 2013), and reduced power during movement is often referred to as desynchronization. Desynchronized beta-band power has also been observed during motor planning prior to movement onset (Kaiser et al., 2001; Alegre et al., 2003; Kilner et al., 2005; Tzagarakis et al., 2010), and during both executed and imagined movement (Pfurtscheller and Lopes da Silva, 1999; Engel and Fries, 2010; Kilavik et al., 2013; Brinkman et al., 2014; Nakayashiki et al., 2014; Meirovitch et al., 2015; Ofori et al., 2015). Clinically, patients with PD who have bradykinesia exhibit abnormal movement-related beta-band cortical oscillations when using either electroencephalography (EEG) or magnetoencephalography (MEG) (Brown and Marsden, 1999; Heinrichs-Graham et al., 2014b; Stegemöller et al., 2016). However, it is not known how beta-oscillations in the cortex change during a ballistic movement, and if the level of desynchronization of cortical neurons relates directly to movement velocity in patients with PD off and on levodopa therapy.

Another key question that is not clear in the literature is how beta-band oscillations in the cortex relate to functional activity in the basal ganglia. This is a difficult question to address as it requires an in vivo approach. A key study found that levodopa reduced beta-band power in the local field potentials (LFP) in the STN, which was associated with the improvement of bradykinesia and rigidity (Kühn et al., 2006). However, this approach does not allow for assaying the relation between basal ganglia and cortical oscillations. The approach taken in the current study uses measures of functional activity in nuclei of the basal ganglia from functional magnetic resonance imaging (fMRI) in relation to how cortical oscillations change with levodopa therapy in patients with PD. It is well established that levodopa is an effective therapy for patients with PD (Jankovic and Aguilar, 2008), and levodopa improves movement velocity of both upper and lower limbs (Baroni et al., 1984; Vaillancourt et al., 2004, Vaillancourt et al., 2006). We use this prior knowledge to examine how levodopa affects cortical oscillations during an upper limb movement. Next, we use a well-established fMRI motor task to measure functional activity in the basal ganglia to compare with the cortical oscillations from EEG. The fMRI motor task has previously shown reduced fMRI activity in the basal ganglia in patients with PD that correlates negatively with motor deficits (Prodoehl et al., 2010), and the fMRI activity in basal ganglia progressively decreases longitudinally in PD (Burciu et al., 2016). Using a multimodal approach with fMRI and EEG allows the examination of functional activity in the basal ganglia and changes in cortical oscillations with levodopa.

A consensus of studies examining STN LFP in patients with PD during DBS have established that both levodopa and high frequency DBS reduce beta-band spectral power in a manner that scales with clinical improvement. This set of findings have led researchers to hypothesize that hypersynchrony of beta-oscillations and pathological oscillations in the basal ganglia may play a prominent role in the pathogenesis of parkinsonism (Little and Brown, 2014). On the other hand, studies of beta-oscillatory activity in the motor cortex have shown variable and sometimes contradictory results regarding the abnormalities that are present in PD, which may be due to heterogeneous modalities and research methods used (Brown and Marsden, 1999; Stoffers et al., 2007; Pollok et al., 2012; Heinrichs-Graham et al., 2014a, Heinrichs-Graham et al., 2014b; Little and Brown, 2014; Moisello et al., 2015; Stegemöller et al., 2016). Here, to examine an association between anti-parkinsonian medication and cortical and subcortical activity in the basal ganglia-thalamo-cortical pathway we used multimodal non-invasive brain imaging techniques to compare patients with PD to healthy controls, and to understand how anti-parkinsonian medication affects brain activity in patients with PD. First, we focused on the initial phase of an isolated ballistic flexion movement of the upper limb in order to evaluate (i) how cortical oscillations are affected by PD during a ballistic movement, (ii) how these oscillations relate to movement velocity, and (iii) the effect of levodopa on cortical oscillatory activity and movement kinematics. Further, we tested the relation between fMRI activity in the basal ganglia and the changes in cortical oscillations to levodopa therapy. To address this question, we used task-based fMRI because this technique has the ability to measure functional activity in the direct and indirect pathway of the basal ganglia nuclei (Spraker et al., 2010). We tested two main hypotheses in this study. The first hypothesis was that an acute dose of levodopa would increase upper limb ballistic movement velocity and voltage of the agonist burst, and modulate beta-band motor cortical oscillations. The second hypothesis was that fMRI activity measured within the putamen and STN during a force control task would predict the modulation of beta-band cortical oscillations by an acute dose of levodopa.

## 2 Materials and methods

### 2.1 Subjects

We studied 15 patients (mean age: 62.00 ± SD 10.87 years; 6 females) and all had a positive response to levodopa as measured by a reduction in the Movement Disorder Society Unified Parkinson's Disease Rating Scale (MDS-UPDRS-III) (*p* < 0.001) (Table 1). Patients with PD were diagnosed by a movement disorder specialist using established criteria (Hughes et al., 2001), and were recruited from University of Florida Center for Movement Disorders and Neurorestoration. 15 age-and sex matched healthy controls (HC; mean age: 62.53 ± SD 8.44 years; 6 females) were also tested. A two-tailed independent *t*-test showed no significant age differences between groups (i.e. PD and HC) (*p* > 0.05) (Table 1). All subjects were asked to refrain from consuming caffeine and refrain from using any hair products on the day of electroencephalography (EEG) and fMRI testing. The experiment was approved by the Institutional Review Board, and all subjects completed an informed consent prior to participating in the study.

**Table 1:** Data are count or mean (±SD). Disease duration is defined as time since diagnosis. Abbreviations: F = females; L = left; LEDD = levodopa equivalent daily dose; M = males; MoCA = Montreal Cognitive Assessment; MDS-UPDRS-III = the motor section of the Movement Disorder Society Unified Parkinson's Disease Rating Scale; mg = milligram; MVC = maximum voluntary contraction; N = Newtons; R = right; yrs. = years.

Demographics|clinical data	Parkinson's disease		Healthy control	Paired T-test	Group T-test
	OFF-medication	ON-medication		*p*-Value	*p*-Value
Sample size	15		15		
Age, yrs	62.00 (10.51)		62.53 ± 8.44		0.931
Gender (M|F)	9 | 6		9|6		
Handedness (L|R)	3 | 12		3|12		
MDS-UPDRS-III – Total	16.80 (8.51)	13.13 (6.98)	1.6 (±1.72)	<0.001	<0.001
MoCA	27.20 (2.18)	27.67 (1.54)	27.80 (±2.01)	0.301	0.574
Total LEDD, mg	649.00 (378.93)				
MVC, N	66.93 (22.71)				
Disease duration, yrs	5.17 (4.78)				
Hoehn and Yahr stage (OFF)	1.67 (0.62)				
More affected side (L|R)	7|8				

### 2.2 Experimental design

**Fig. 1:**
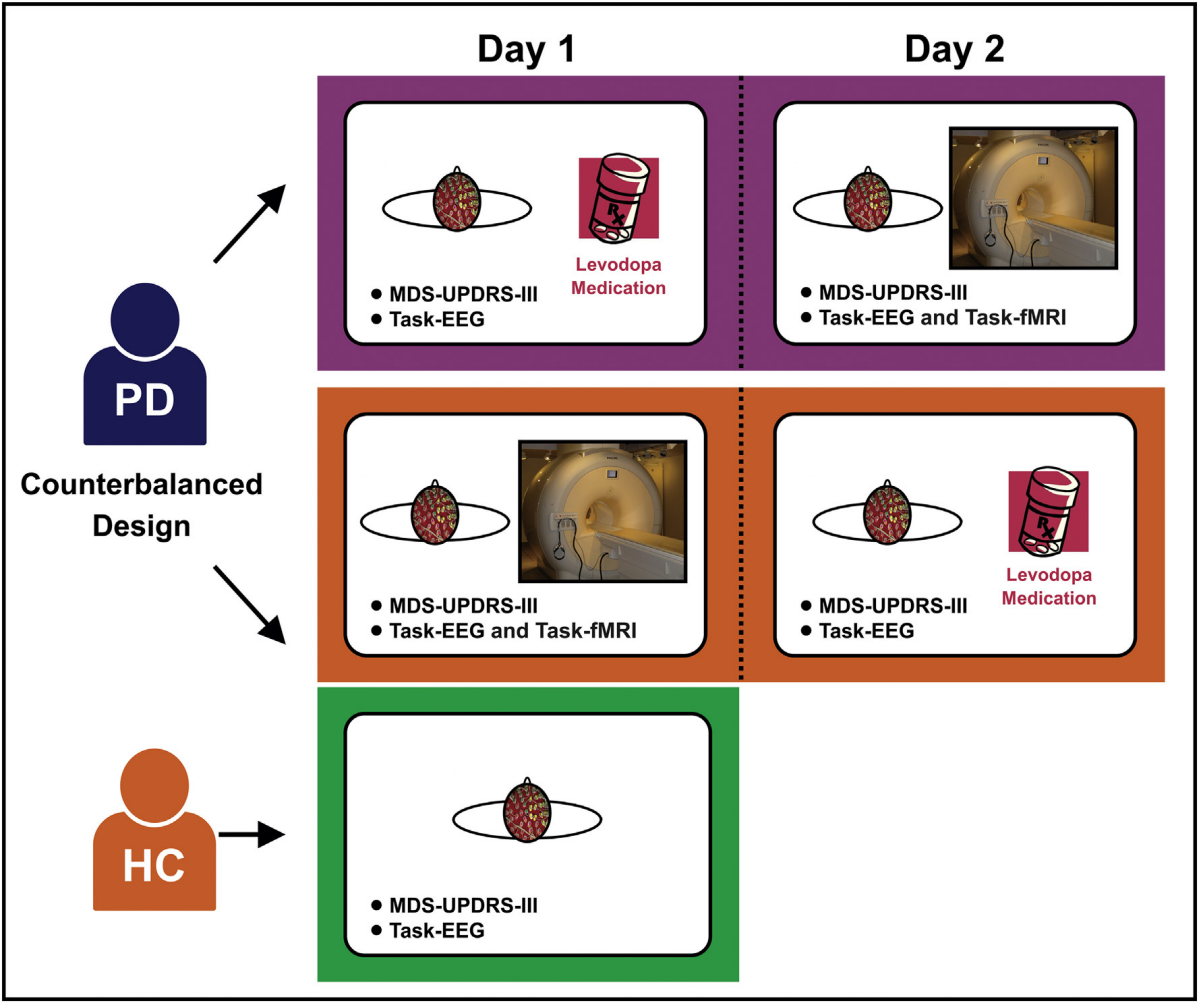
Experimental design schema. There are two groups (i.e. patients with Parkinson's disease (PD) and healthy controls (HC)) in this study. The experiments for the PD group were performed on two consecutive days (i.e. the on medication day and off medication day). The order of testing medication across days was counter-balanced across PD group. Each patient with PD underwent high-density EEG and assessment of motor symptoms (MDS-UPDRS-III) in both days. In addition, patients with PD were studied using task-based fMRI in the on medication day. The experiments of HC were included EEG and MDS-UPDRS-III. Abbreviations: EEG = electroencephalography; fMRI = functional magnetic resonance imaging; MDS-UPDRS-III = the motor section of the Movement Disorder Society Unified Parkinson's Disease Rating Scale; PD = Parkinson's disease.

The experiments for patients with PD were performed on two consecutive days: (i) a functionally off medication day (PD-OFF) and (ii) on dopaminergic medication (levodopa) day (PD-ON). The order of testing (PD-OFF and PD-ON) across days was counter-balanced across patients with PD. PD-OFF testing occurred in the practically defined PD off medication state (Langston et al., 1992), with testing following at least 12-hour withdrawal from dopaminergic medication. PD-ON testing occurred approximately 45 min after taking the usual morning dose of medication, corresponding to the time required for levodopa plasma level to reach its peak (Brooks, 2008). Each patient with PD underwent high-density EEG and assessment of motor symptoms and cognitive status during both PD-OFF and PD-ON. Motor symptoms and cognitive status were assessed using MDS-UPDRS-III and the Montreal Cognitive Assessment (MoCA), respectively (Nasreddine et al., 2005; Goetz et al., 2008). In addition, patients with PD were studied using task-based fMRI on the PD-OFF day. HC underwent the same EEG, MDS-UPDRS-III, and MoCA protocol once (Fig. 1).

### 2.3 EEG experimental task

**Fig. 2:**
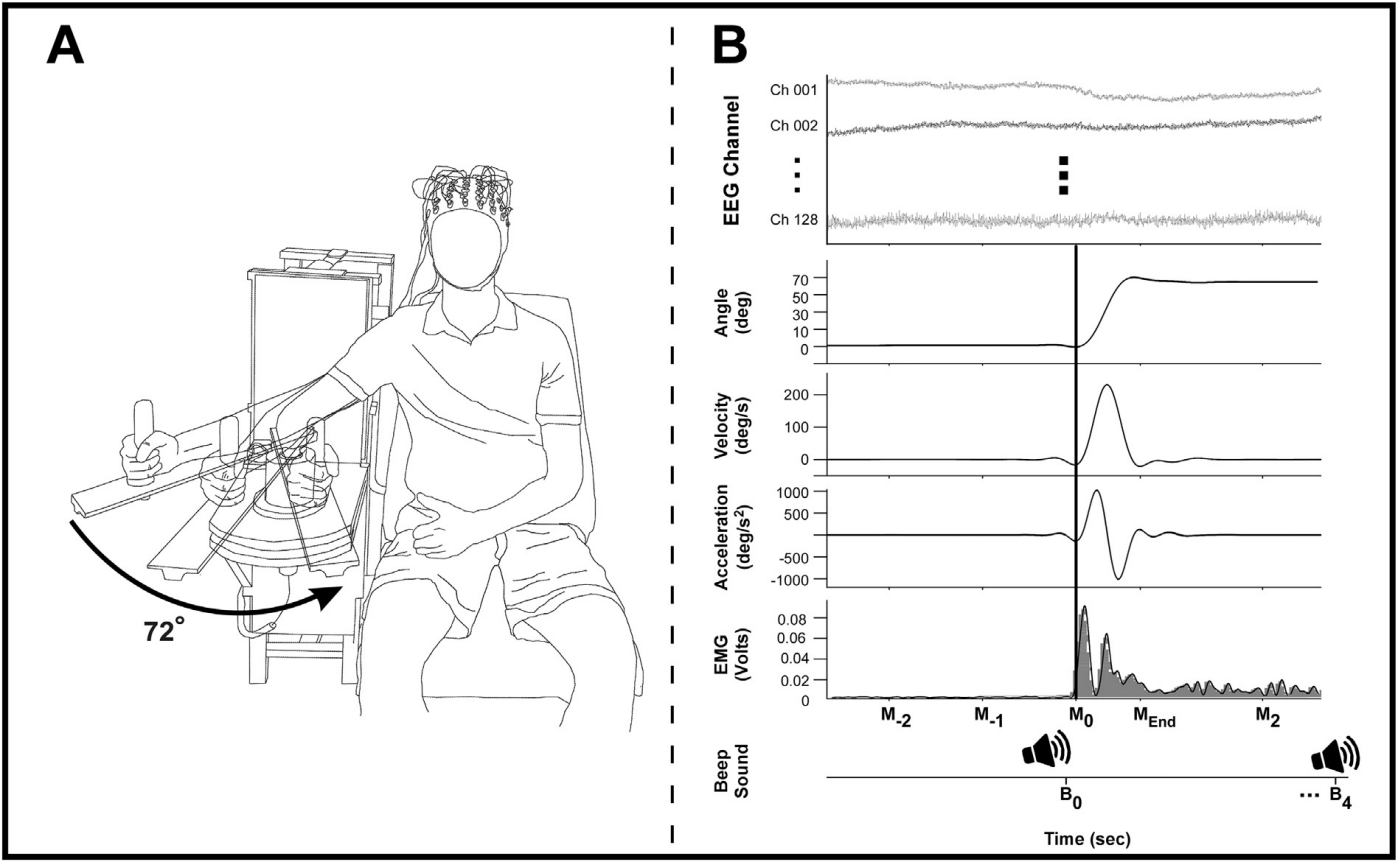
Experimental setup of the task, example kinematic and neuromuscular data. Fig. 2A shows a schematic of the experimental set-up. Fig. 2B depicts examples of analyses performed on the EEG, kinematic, and EMG records using data from an individual movement trial, and shows EEG channels, movement displacement, velocity, and acceleration, and biceps agonist EMG. The bottom section of Fig. 2B shows the Beep Sound time line, depicting when subjects were asked to move their arm to the target (B_0_) and when subjects were asked to return to the start position (B_4_). The black vertical line is aligned on the movement on-set time (M_0_) across multiple data. Abbreviations: B_0_ = the first beep sound to move subjects' arm; B_4_ = the second beep sound to return subjects' arm to the start position from the target (4 s after the first beep sound); M_0_ = the movement onset time (5% of the peak acceleration); M_End_ = the movement termination time; M_2_ = 2 s after the movement onset time; M_−1_ = 1 s before movement onset; M_−2_ = 2 s before movement onset.

For the EEG paradigm, we used an upper limb ballistic movement task that was similar to prior work (Vaillancourt et al., 2004; Ofori et al., 2015). We asked subjects to sit upright in a chair with their right arm supported against a cantilever beam attached to a custom-made manipulandum (Fig. 2A) and to move their right arm in the horizontal plane “as fast and accurately as possible” by flexing the elbow to the target angle (72°). We provided visual feedback of the subject's arm angular position to the subject through a 76.2 Cm monitor screen (Dell UltraSharp U3011, Dell Co, Round Rock, TX) that displayed a starting position, target position, and a cross-shaped cursor that showed the arm angular position. The starting position (0°) was represented as a stationary bar on the right middle of the monitor and the target position (72°) was set to 42 cm to the left of the start position (0°). The time allotted for each trial was 12 s. After a 5 s baseline in which the subject's right arm was at the starting position (0°), a 400 Hz auditory beep cue signaled subjects to begin the movement (Fig. 2B). During the subsequent 4 s movement period, subjects were asked to use their right elbow flexors to move the manipulandum to the target position (72°) as fast and accurately as possible from the starting position (0°) and keep its position there. A second auditory beep cued the final 3 s period in which subjects were instructed to return the cursor (and arm) to the starting position (0°) from the target position (72°). We asked patients with PD to perform 50 trials under each medication condition and HC subjects to perform 50 trials.

#### 2.3.1 Kinematic, EMG, and EEG data acquisition

The MotionMonitor (Innovative Sports Training, Inc., Chicago, IL) system was used to synchronize multi-data with different sampling rates (i.e., EEG – 2048 Hz, electromyography (EMG) – 2000 Hz, and kinematic data – 1000 Hz) simultaneously by using a trigger pulse delivered to each data device that time-synced using a common analogue signal.

##### 2.3.1.1 Kinematic data acquisition

The kinematic data were collected with an angular displacement transducer. The transducer was mounted at the axis of rotation of the manipulandum. An excitation voltage of 16 V from a Leader LPS-152 DC Tracking Power Supply (Advanced Test Equipment Rentals, San Diego, CA) was used to power the angular displacement transducer. The displacement data were transmitted via a 16-bit A/D converter and digitized using a USB-1616HS-BNC A/D board (Measurement Computing, Norton, MA).

##### 2.3.1.2 EMG data acquisition

EMG data were collected with the Delsys Trigno Wireless System (Delsys Inc., Boston, MA). Subjects were prepped by rubbing the desired location on the right arm with an alcohol swab and a wireless EMG electrode was placed on the subject's right biceps brachii.

##### 2.3.1.3 EEG data acquisition

EEG data were collected with the ActiveTwo system (Cortech Solution, Wilmington, NC) that was comprised of 128 Ag-AgCl Active Two electrodes. The active electrodes were connected to a head cap that was preconfigured in a 128-electrode montage covering the entire scalp. The head cap size was selected based on the subject's head circumference (50–54, 54–58, or 58–62 cm). The signals were amplified through the electrodes at the source and had an output impedance of <1 Ω. Electrical potentials were recorded between each electrode and Common Mode Sense (CMS) active electrode and a Driven Right Leg (DRL) passive electrode located at the center of the scalp in relation to all the other electrodes. The CMS and DRL electrodes were used to drive the average potential of the subject as close as possible to the AD-box reference potential electrode. The electrode offsets, a running average of the voltage measured between the CMS and each active electrode, were evaluated before the start of each condition and during data collection to be within the acceptable range of ±40 μV. The electrode offset served as an indirect measure of impedance tolerance. To ensure that a stable and high-quality signal was recorded from each active electrode throughout the recording session, the electrode offset was monitored.

#### 2.3.2 Kinematic, EMG, and EEG data processing and analysis

All behavioral and electrophysiological data were exported at 1000 Hz by the MotionMonitor (Innovative Sports Training, Inc., Chicago, IL) system and imported into MATLAB R2014a (The Mathworks, Natick, MA).

##### 2.3.2.1 Kinematic analysis

Arm angular position data (i.e., arm angular displacement data) were lowpass filtered at 2 Hz (Butterworth 4th order dual pass). Velocity and acceleration data were calculated based on the differential of the angular position signal. The movement onset was defined by 5% of the peak acceleration (Ofori et al., 2015; Chung et al., 2017). Kinematic data were comprised of peak displacement and peak velocity during the movement.

##### 2.3.2.2 EMG analysis

EMG data were highpass filtered at 1 Hz (Butterworth 4th order dual pass) to remove the DC offset, rectified and lowpass filtered at 10 Hz (Butterworth 4th order dual pass). Integrated EMG (iEMG) was computed across the movement initiation phase from 20 ms before the movement onset to the peak acceleration time.

##### 2.3.2.3 EEG preprocessing

EEG data were processed using custom routines based on EEGLAB used in previous work (Ofori et al., 2015; Chung et al., 2017). First, EEG data were bandpass filtered between 1 and 70 Hz. The mean SD was calculated by averaging the SDs of all channels. Any channel with a SD > 2 times the mean SD was rejected and then interpolated from the spherical neighboring channels. The EEG signals were then re-referenced to the global average of all 128-EEG channels. For removing sinusoidal artifacts (60 and 120 Hz line noise) we used the CleanLine function within EEGLAB (Bigdely-Shamlo et al., 2015). EEG data epochs were extracted from 1100 ms before movement onset to 900 ms seconds after movement onset for all trials. EEG data were prepared for further analysis by concatenating all epochs within each condition within each subject. Joint probability was calculated to find outlier epochs with improbable activity from each subject and each condition (Delorme et al., 2007). The concatenated data were screened by using a joint probability threshold SD equal to 5.

##### 2.3.2.4 EEG source localization

The screened EEG data were decomposed using independent component analysis (ICA). The Infomax ICA algorithm (Bell and Sejnowski, 1995) with the natural gradient feature (Amari et al., 1996) was employed to decompose the concatenated EEG data of each subject into maximally ICs. Source localization of each IC was performed using the DIPFIT function within EEGLAB, which computes an equivalent current dipole model that best explains the scalp topography of each IC. Each dipole was identified through a finite element spherical head model from Brain Electrical Source Analysis (BESA GmbH, Gräfelfing, Germany). The BESA head model has a template channel location map based on the standard 10–5 system. The actual channel location map was mapped onto the BESA template channel location map, which in turn was co-registered to the Montreal Neurological Institute (MNI-152) brain template. ICs were excluded if the projection of the equivalent current dipole to the scalp accounted for >15% of the residual variance, was outside of the brain, or if the time-course, spectra, and topography of ICs were reflective of eye movement or electromyography artifact (Ofori et al., 2015; Chung et al., 2017).

##### 2.3.2.5 EEG measure projection analysis

Measure projection analysis (MPA) is a statistical method for characterizing the localization and consistency of EEG measures across sessions of EEG recordings (Bigdely-Shamlo et al., 2013). It allows the use of high density EEG as a 3D cortical imaging modality with near-cm scale spatial resolution. MPA identifies domains and defines anatomical regions of interest (ROIs) and finds ratios of domain masses for cortical structures which incorporates the probabilistic atlas of human cortical structures provided by the Laboratory of NeuroImaging (LONI) project 3D probabilistic atlas of human cortical structure (Shattuck et al., 2008).

This procedure clusters domains based on measure specific data from all individual subject IC locations. These domains are identified in a data-driven manner with unique EEG measure (e.g., ERSP) time-frequency features in 3D space. To create the domains, a cubic space grid with 8-mm spacing was situated in the brain volume in MNI space that served as the MPA brain model. Voxels outside the brain model were excluded. Local convergence values were calculated based on the algorithm explained in detail by Bigdely-Shamlo et al. (2013). Local convergence helps deal with the multiple comparisons problem by finding measure similarity of dipoles and comparing them with randomized dipoles. A pair wise IC similarity matrix was constructed by estimating the signed mutual information between IC-pair event-related power spectral perturbation (ERSP) measure vectors using a Gaussian distribution assumption. Signed mutual information was estimated to improve the spatial smoothness of obtained MPA significance values (Bigdely-Shamlo et al., 2013). A significance threshold for convergence at each brain location was obtained by bootstrap statistics. The raw voxel significance threshold was set to *p* < 0.05, which is based on prior studies in the literature (Bigdely-Shamlo et al., 2013; Ofori et al., 2015).

Within each domain, ERSPs were computed for each group (HC vs PD-OFF) or each condition in PD (PD-ON vs PD-OFF) to identify region-specific and frequency-specific oscillatory activity during the movement. In the ERSP, the baseline was set to 100 ms before movement onset. The baseline period was then 1 s before baseline, and the active period was 1 s after baseline. The decibel (dB) scale unit was used for time-frequency power normalization and denoted by the following equation:

$${\mathrm{dΒ}}_{tf}=10\;\log10\left(\frac{{\mathrm{activity}}_{tf}}{{\bar{\mathrm{baseline}}}_{f}}\right)$$

where $\bar{\mathrm{baseline}}$ is the mean across the baseline period, and t and f are time and frequency points (Makeig, 1993). Theta (3–8 Hz), alpha (8–12 Hz), beta (13–30 Hz), and gamma (31–50 Hz) bands were examined. For each identified domain, significant differences in the power at each frequency band (between HC and PD-OFF groups, and between PD-ON and PD-OFF medication conditions) were computed by projecting the ERSP associated with each condition or each group to each voxel in a domain. This produced a projected measure. Next, a weighted-mean measure across all domain voxels was weighted by the dipole density of an individual voxel per subject. The measure was then normalized by the total domain voxel density. Analysis of projected source measures were separated into distinct spatial domains by threshold-based Affinity Propagation clustering based on a similarity matrix of pair-wise correlations between ERSP measure values for each position. For the current study, the maximal exemplar-pair similarity, a parameter that ranges from 0 to 1.0 was set to a value of 0.8 based on prior work (Bigdely-Shamlo et al., 2013; Ofori et al., 2015; Chung et al., 2017).

The beta-band power was computed for each subject for each domain over the baseline period. For patients with PD, the beta-band power was computed separately for each condition (PD-OFF and PD-ON). To establish the beta-band power for a subject in a given domain, we first averaged the IC source time series of dipoles constituting the domain. Then we estimated the spectrum of the averaged signal and computed the power in the beta-band.

### 2.4 fMRI experimental task

For the task-based fMRI paradigm performed in the PD-OFF medication condition, we used a force control paradigm that required patients to perform a pinch force task with their more affected hand (defined by the upper limb items from the MDS-UPDRS-III scale). Previous studies have shown the BOLD signal of fMRI during a force control task is reduced in PD compared with control individuals (Spraker et al., 2010; Burciu et al., 2016). Prior to entering the MRI scanner, patients with PD were trained on the task. At the beginning of the training session, each patient with PD's maximum voluntary contraction (MVC) was measured using a Jamar hydraulic pinch gauge over three trials. The corresponding output values were used to compute an average MVC that served to normalize force data across patients with PD. For each patient with PD, the target force level was set at 15% of the MVC. The task-fMRI protocol consisted of a block-design that alternated force and rest blocks as follows: 30 s rest, 30 s force with performance feedback, 12.5 s rest, and 30 s force without performance feedback (Prodoehl et al., 2010; Spraker et al., 2010; Planetta et al., 2014; Burciu et al., 2015; Neely et al., 2015; Burciu et al., 2016). This sequence was repeated four times and there was an additional 30-s rest period following the final block of force without performance feedback. The scan lasted 7 min and 33 s. Throughout the scan, two bars were displayed on an LCD monitor that patients could see through a mirror mounted on the MRI head coil: a target bar and a force bar. A change in color of the force bar cued patients with PD to either push or release the force sensor. Green was a go-signal for rapidly producing and sustaining force (2 s) at the target bar (15% MVC), while red indicated a rest period (1 s) to release force. Force was produced in the presence of performance feedback as well as in the absence of performance feedback (four blocks per condition). In the feedback condition, the target bar was stationary at 15% MVC, while the force bar moved in the vertical plane according to the force output. Instructions were to produce force in order to bring the force bar on top of the target bar for each 2 s period. In the no-feedback condition, both target and force bars were stationary. Patients with PD were required to produce and maintain a 15% MVC force during each 2 s period without feedback, and the timing of the force contractions was controlled by the same green and red bars.

#### 2.4.1 fMRI Acquisition

##### 2.4.1.1 MRI acquisition

MRI was performed on a 3T system (Philips Achieva) equipped with a 32-channel quadrature volume head coil. Head movement was minimized by foam padding within the coil and scanner noise was attenuated using a combination of earplugs and circumaural headphones. Functional images were obtained using a T_2_*-weighted, single shot, echo-planar pulse sequence with the following parameters: repetition time = 2500 ms, echo time = 30 ms, flip angle = 80^o^, field of view = 240 mm^2^, acquisition matrix = 80 × 80, voxel size = 3 mm isotropic with no gap between slices (n = 46). A 3D T_1_-weighted image was collected: repetition time = 8.2 ms, echo time = 3.7 ms, flip angle = 8^o^, field of view = 240 mm^2^, acquisition matrix = 240 × 240, voxel size = 1 mm isotropic with no gap between slices (n = 170). All scans were acquired axially.

##### 2.4.1.2 fMRI force task acquisition

Force data were collected with an MRI compatible force transducer with a resolution of 0.025 N (Neuroimaging Solutions LLC, Gainesville, FL). The analog force data produced by the patient was transmitted with a fiber-optic cable to a SM130 Optical Sensing Interrogator (Micron Optics, Atlanta, Georgia), which digitized force data at 125 Hz. Custom software in LabVIEW (National Instruments, Austin, TX) converted the digitized force data to Newtons. The output was presented to the subject using a visual display as described.

#### 2.4.2 fMRI data analysis

Consistent with previous studies, the fMRI and T_1_-weighted scans of those subjects who performed the task with their left hand were flipped along the midline prior to preprocessing (Prodoehl et al., 2010; Spraker et al., 2010; Planetta et al., 2014; Burciu et al., 2015; Neely et al., 2015; Burciu et al., 2016). Data analysis was performed using Analysis of Functional NeuroImages (AFNI) and included the following steps: 1) removal of the first four volumes of the functional scan to allow for T_1_ equilibration effects, 2) slice timing and head motion correction, 3) normalization of the signal in each voxel at each time point by the mean of its time series, 4) registration of each volume of the functional data set to its first volume, 5) co-registration of the functional scan with the structural scan, 6) spatial normalization of the structural scan to the MNI_152_ template, 7) reslicing of the functional scan in MNI space using the normalization parameters from the previous step, and 8) smoothing of the functional scan with a Gaussian kernel of 4 mm full width at half-maximum (FWHM). Finally, task-fMRI data were regressed to a simulated hemodynamic response function for the task sequence (using the 3Ddeconvolve function in AFNI). Next, a mean percent signal change was calculated for a 15 s period (6 TRs out of 12) (Burciu et al., 2016) toward the end of the force block in pre-specified regions of interest (ROIs) that were selected based on prior studies showing reduced BOLD activity in PD compared to HC (Spraker et al., 2010; Burciu et al., 2016). The pre-specified ROIs consisted of the contralateral (defined with respect to the hand tested) putamen and STN and were extracted from the Basal Ganglia Human Area Template (BGHAT) (Prodoehl et al., 2008).

### 2.5 Statistical analysis

After EEG data processing, one of 15 patients with PD was excluded due to IC dipoles failing the DIPFIT model. For the kinematic variables and iEMG, an independent *t*-test was conducted between groups (HC (n = 15) vs. PD-OFF (n = 14)) and a paired sample *t*-test was used for medication condition comparisons within PD (n = 14) (PD-ON vs. PD-OFF). For EEG ERSP measures, a non-parametric bootstrapped independent *t*-test was performed to compare groups (HC (n = 15) vs. PD-OFF (n = 14)) at each domain and a non-parametric bootstrapped paired sample *t*-test was used to compare medication conditions within PD (n = 14) at each domain. *p*-values were then false discovery rate (FDR) corrected to *p* < 0.05, controlling for multiple comparisons across the time-frequency matrix (Benjamini and Hochberg, 1995). For beta-band power of EEG ERSP measures at baseline, an independent sample *t*-test and a paired *t*-test at each domain were conducted. We performed separate regression analyses to evaluate if beta-band spectral power at each domain predicted the peak velocity in PD. Beta-band (13–30 Hz) ERSP values were extracted in the 13–30 Hz range and averaged from movement onset to 150 ms after onset for each patient with PD and each condition at each domain. To determine if BOLD activity in nodes of the basal ganglia predicted the medication-response in the cortex, we performed separate regression analyses examining the difference in beta-band spectral power before and after levodopa (PD-ON vs PD-OFF) at each domain (dependent variables) and percent signal change in contralateral putamen and STN in PD-OFF state (independent variables).

## 3 Results

### 3.1 Kinematic data

**Fig. 3:**
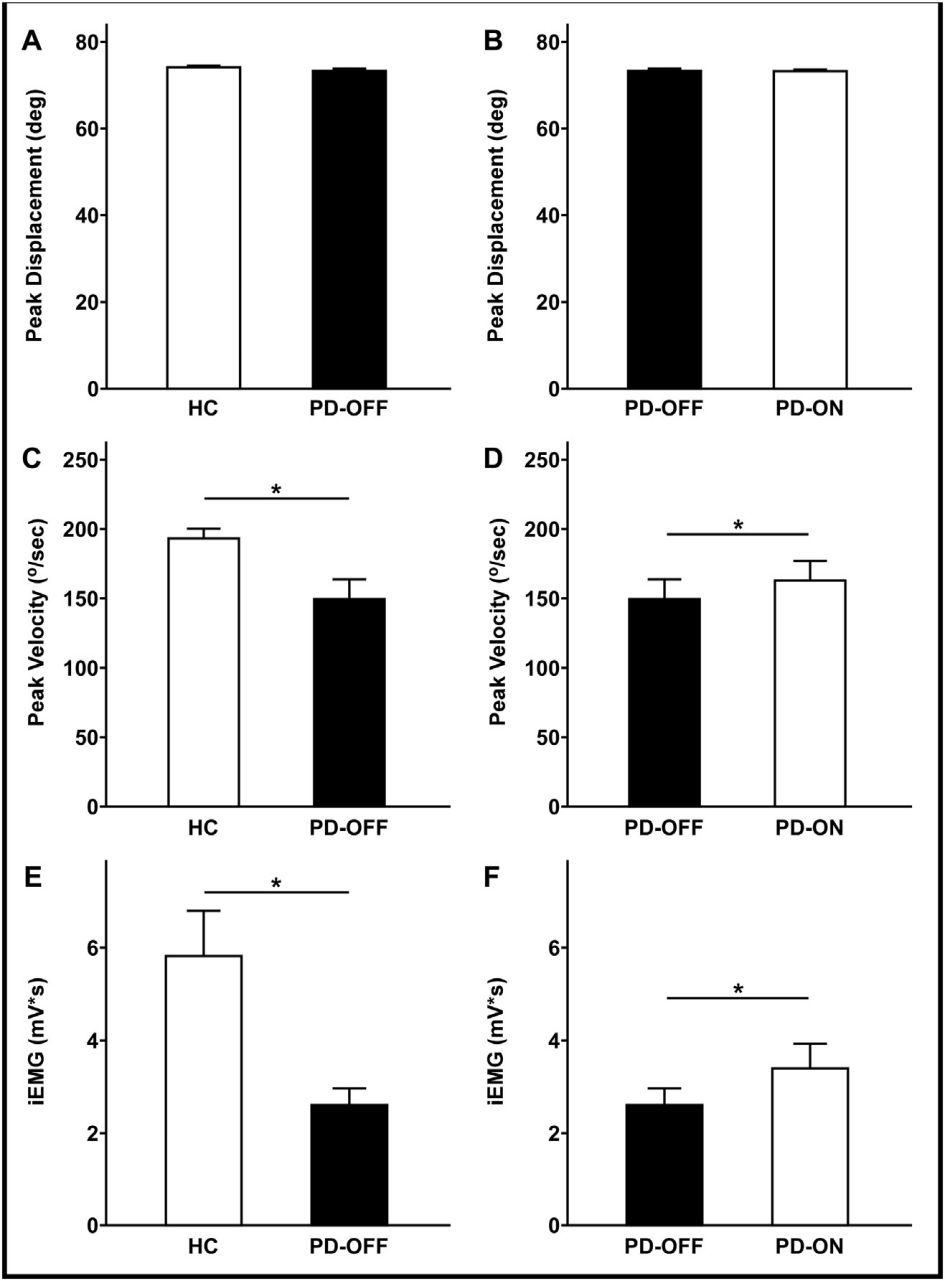
Bar graphs for the task performance variables. Fig. 3A and B show bar graphs of average peak displacement for group comparison and medication condition in the patient group. Fig. 3C and D show bar graphs of average peak velocity and Fig. 3E and F show bar graphs of average integrated EMG (iEMG) in the movement initiation phase. Asterisks indicate significant group effects or medication effects at FDR-corrected *p*-value < 0.05. All error bars represent standard error (N_HC_ = 15, and N_PD_ = 15). Abbreviations: iEMG = integrated EMG, PD-ON = PD taking levodopa; PD-OFF = PD off levodopa overnight.

There was no significant group effect (PD-OFF vs. HC; *p* = 0.201) or effect of dopaminergic medication (PD-ON vs. PD-OFF; *p* = 0.844) on the peak displacement during the ballistic upper limb movement (Fig. 3A and B). HC showed greater movement velocity than patients with PD tested off medication (*p* = 0.013). After administration of dopaminergic medication, patients with PD moved at a greater velocity than in the off medication state (*p* = 0.044) (Fig. 3C and D).

### 3.2 EMG data

Fig. 3E and F show the integrated EMG (iEMG) on the right biceps brachii during the movement initiation phase (i.e., from 20 ms before the movement onset to the peak acceleration time). HC showed greater iEMG than PD-OFF (*p* = 0.006). iEMG was increased in PD-ON during movement initiation in patients with PD (*p* = 0.022) (Fig. 3E and F).

### 3.3 EEG data

**Fig. 4:**
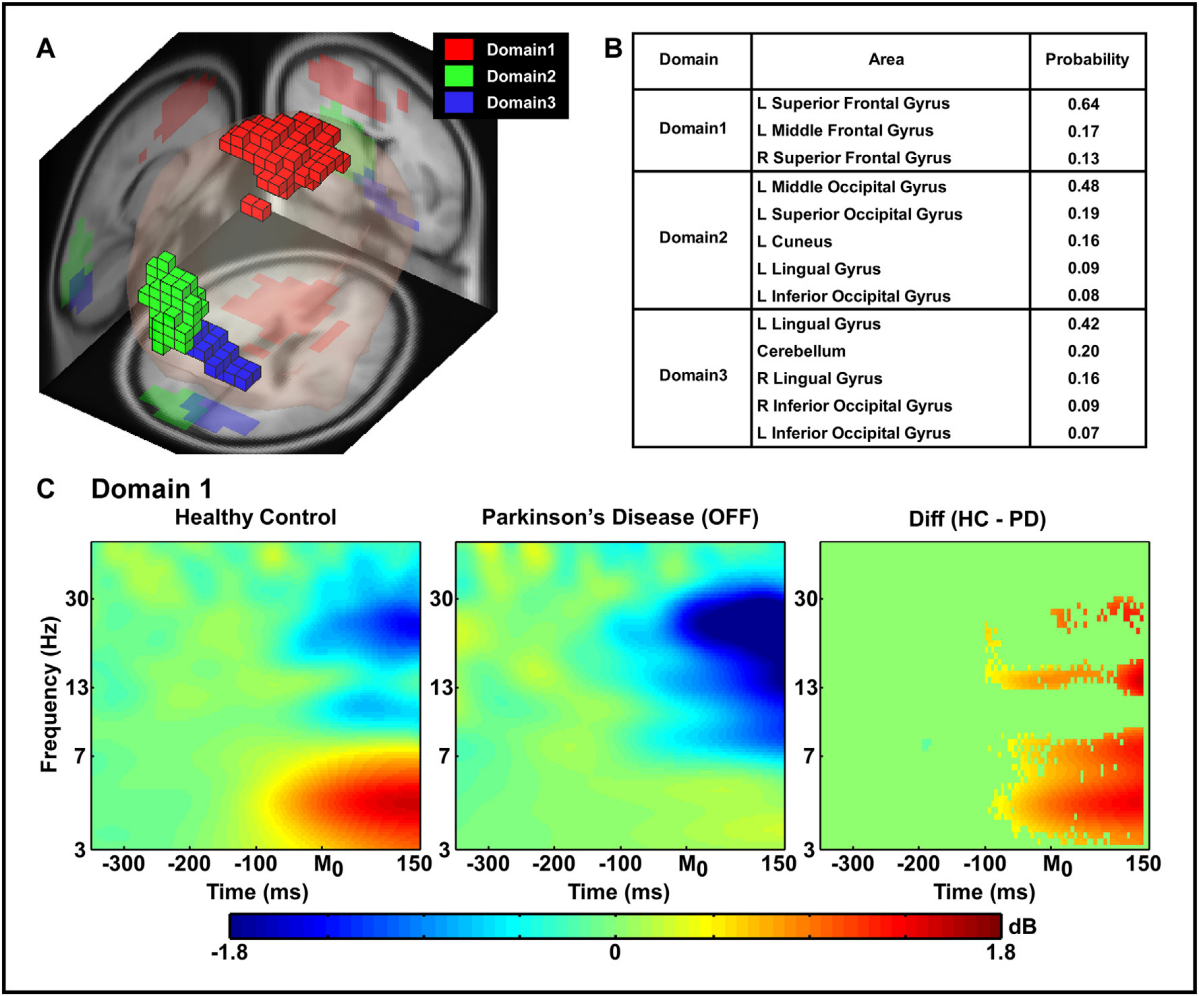
Measure projection analysis (MPA) of the event-related spectral perturbation (ERSP) measure and time-frequency contrast statistical plot (Group comparison between healthy control and PD groups). Fig. 4A displays 3D representation of the brain area of ERSP domains on the MNI brain template. Fig. 4B shows anatomical localization of each domain. Fig. 4C shows ERSP measures from the supplementary motor area (i.e. Red blocks, Domain 1 in Fig. 4A) on each group and time-frequency contrast statistical plot. The x-axis denotes time (ms) with M_0_ representing movement onset, −300 representing 300 ms before movement onset and 150 representing 150 ms after movement onset. The logarithmic y-axis depicts frequencies from 3 to 50 Hz. The plots are color scaled based on decibel where positive values are warm colors and negative values are cool colors. In the right plot which shows statistical group differences, red color indicates statistically greater decibel values (i.e. HC > PD) whereas blue indicates lower decibel values (i.e. HC < PD). The green regions indicate non-significant areas of the plot. The statistical results were FDR corrected *p* < 0.05.

**Fig. 5:**
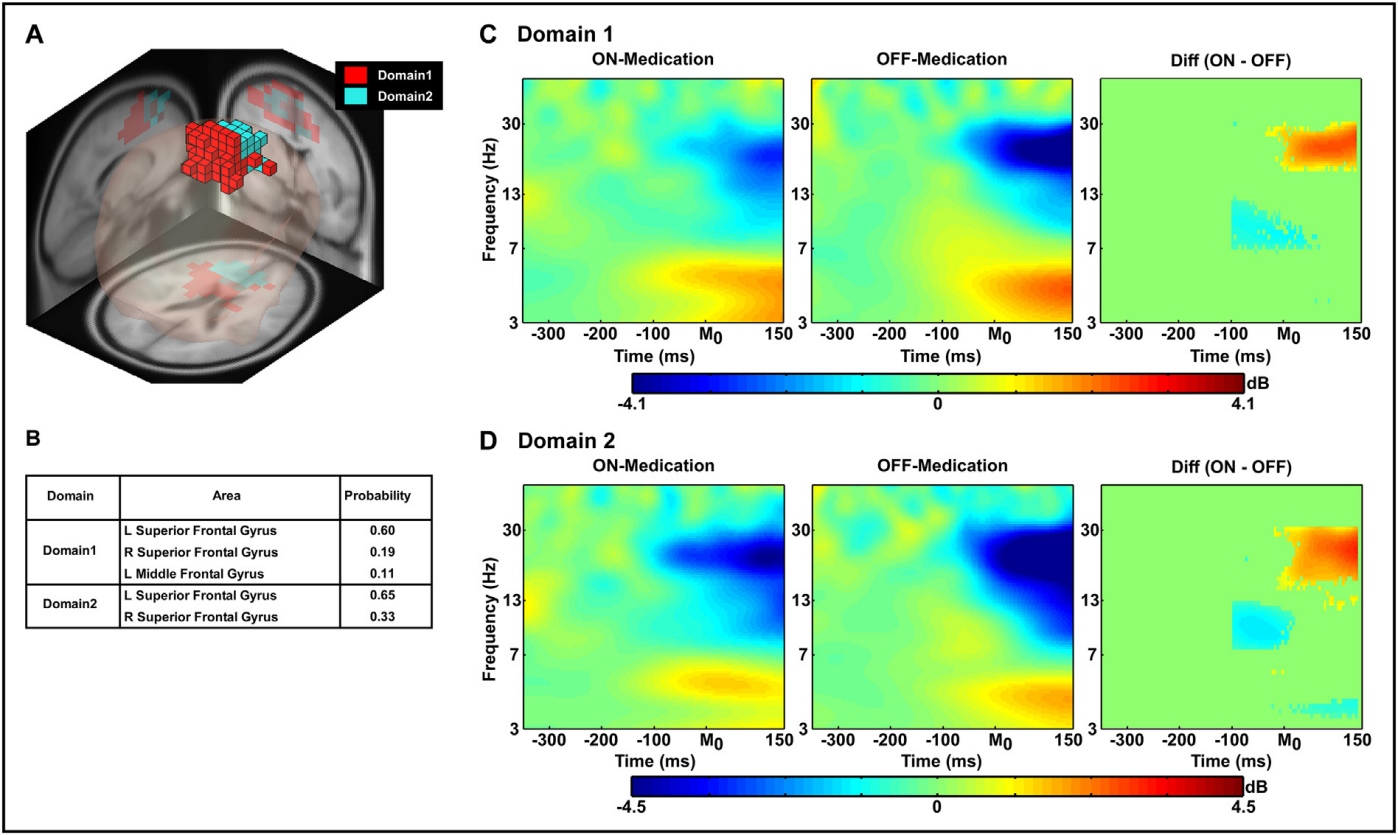
Measure projection analysis (MPA) of the event-related spectral perturbation (ERSP) measure and time-frequency contrast statistical plot (levodopa medication condition comparison in the PD group). Fig. 5A displays 3D representation of the brain area of ERSP domains on the MNI brain template. Fig. 4B shows anatomical localization of each domain. Fig. 4C and D show ERSP measures from Domain 1 and Domain 2 on each condition in PD group and time-frequency contrast statistical plots. The x-axis denotes time (ms) with M_0_ representing movement onset, −300 representing 300 ms before movement onset and 150 representing 150 ms after movement onset. The logarithmic y-axis depicts frequencies from 3 to 50 Hz. The plots are color scaled based on decibel where positive values are warm colors and negative values are cool colors. In the right plot which shows statistical levodopa medication condition differences in the PD group, red color indicates statistically greater decibel values (i.e. ON-Medication condition > OFF-Medication condition) whereas blue indicates lower decibel values (i.e. ON-Medication condition < OFF-Medication condition). The green regions indicate non-significant areas of the plots. The statistical results were FDR corrected *p* < 0.05.

Fig. 4, Fig. 5 show the common source domains identified by MPA and the ERSP time-frequency contrasts for the domains that were significantly different between groups or medication state. For each figure, section A shows the domains identified by MPA, section B shows the corresponding anatomical areas with probability, and section C shows the ERSP time-frequency plots and the difference in ERSP for each significant domain after statistical testing (independent *t*-tests on ERSP between groups and paired *t*-tests on ERSP between dopaminergic medication conditions).

Fig. 4 shows the results of the comparison between HC and PD-OFF groups. MPA identified the supplementary motor area (SMA; Domain 1) and two visual cortex domains (Domains 2 and 3) during the task. Statistical analysis revealed that in the SMA (Domain1), PD-OFF had an exaggerated degree of movement-related desynchronization (i.e. reduction in spectral power before and shortly after movement onset) in the beta-band and reduced movement-related synchronization in the theta band compared to HC starting 100 ms before movement onset [FDR *p*'s < 0.05]: ERSP in Domains 2 and 3 did not differ between groups.

Fig. 5 shows the results of the comparison between PD-ON and PD-OFF medication states. MPA identified two distinct domains within the SMA (Domains 1 and 2). Statistical analysis revealed that PD-ON had a reduction in movement-related beta-band desynchronization compared to PD-OFF after movement onset in both SMA domains [FDR *p*'s < 0.05]. In the alpha-band, however, PD-ON had a greater degree of movement-related desynchronization than PD-OFF 100 ms before movement onset in both SMA domains. In SMA Domain 2, PD-ON showed less movement-related theta-band synchronization (i.e. less spectral theta-band power) than PD-OFF after movement onset.

We conducted independent samples t-test for the baseline beta-band power. We did not observe a significant difference in beta-band power at baseline between groups (HC vs PD-OFF) (SMA Domain 1: *p* = 0.211), and this is likely due to the large between subject variability of the control group. We also conducted a paired t-test comparing off and on medication for patients with PD in each domain. We did not observe a significant difference in beta-band power at baseline between medication conditions (SMA Domain 1: *p* = 0.127, and SMA Domain 2: *p* = 0.124).

### 3.4 Regression analysis

**Fig. 6:**
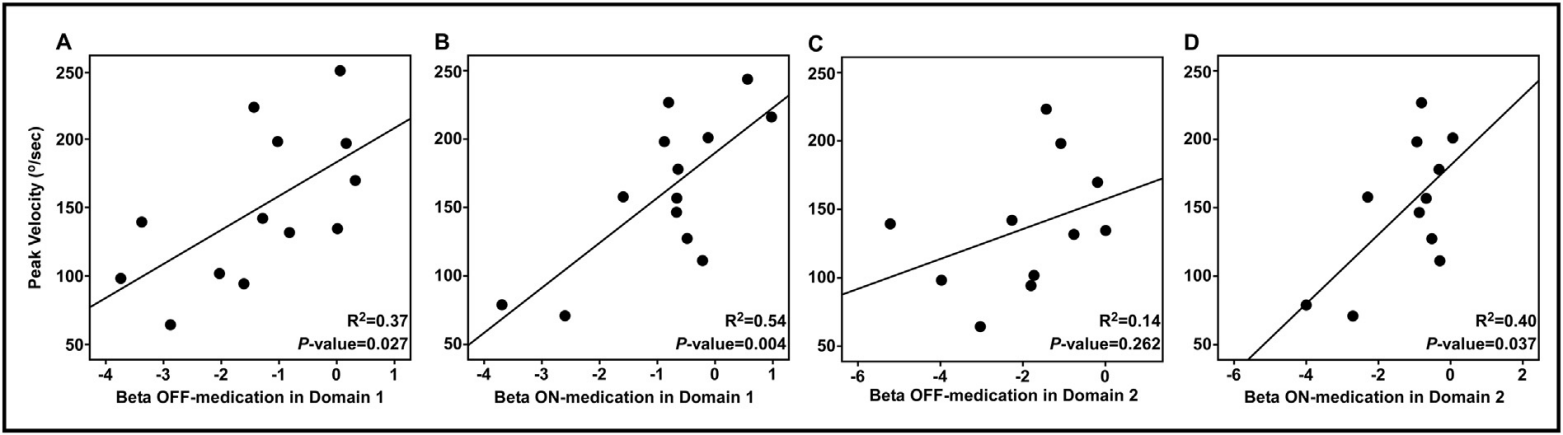
Linear regression between average peak velocity during the task and beta-band spectral power after movement onset in the PD group. Fig. 6A and B show the linear regression for 13 patients with PD in Domain 1. Fig. 6C and D show the linear regression for 11 patients with PD in Domain 2. Fig. 6A and C are PD group off medication and Fig. 6B and D are PD group on medication.

Fig. 6 shows the association between the peak velocity of movement during the task and spectral power in the beta-band after the movement onset in the PD group. In Domain 1 (see Fig. 6A and B), both PD off medication and PD on medication showed that increased beta-band spectral power led to an increase in the peak velocity of movement (PD-OFF in Domain 1: (F(1, 11) = 6.521, *p* = 0.027; PD-ON in Domain 1: (F(1, 11) = 13.099, *p* = 0.004). In Domain 2 (see Fig. 6D), only PD on medication showed that higher beta-band spectral power was associated with faster movement; (PD-OFF in Domain 2: (F(1, 9) = 1.430, *p* = 0.262; PD-ON in Domain 2: (F(1, 9) = 5.955, *p* = 0.037).

**Fig. 7:**
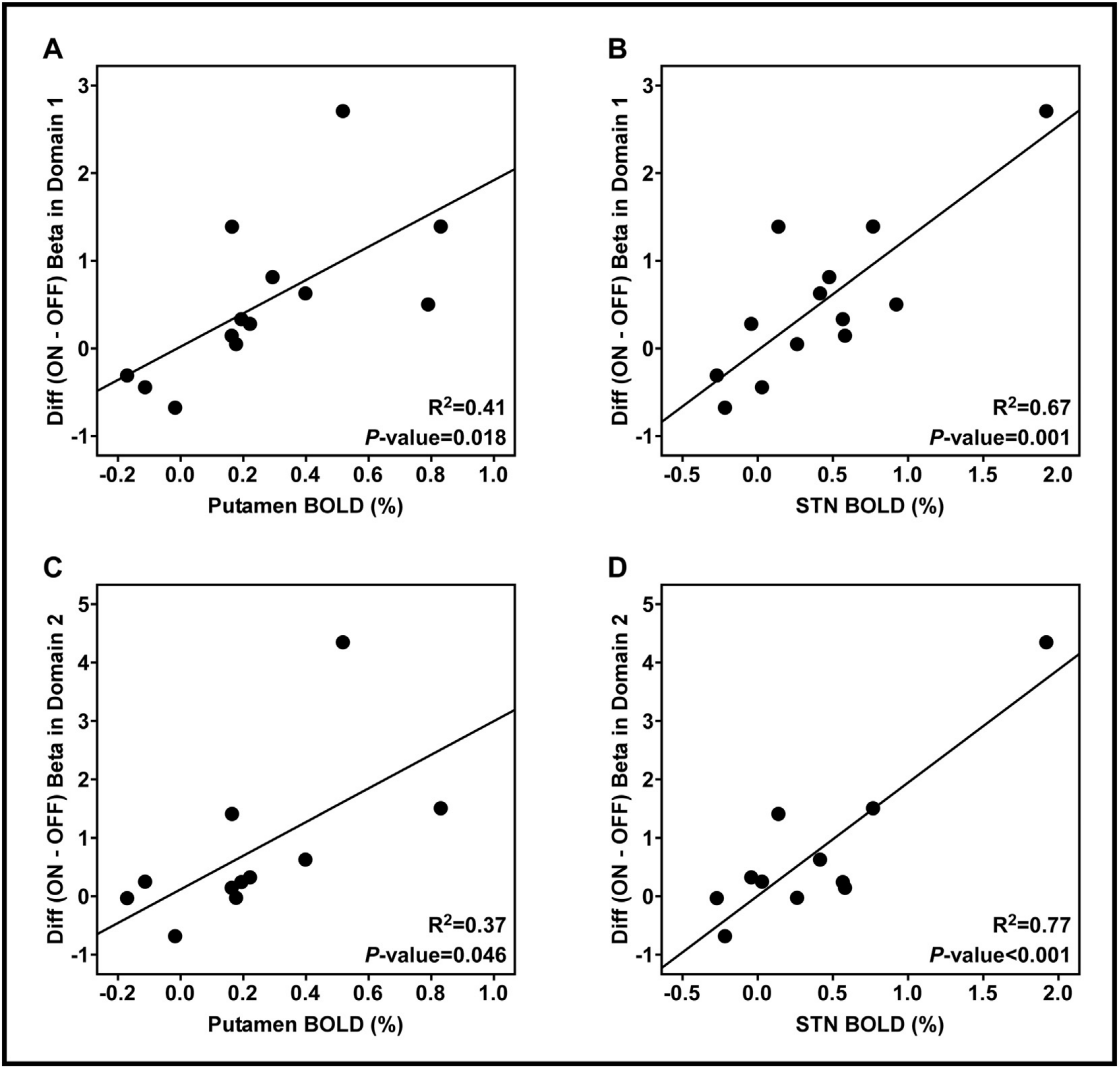
Linear regression between fMRI BOLD signals in both the contralateral putamen and STN and the differences between levodopa medication conditions in the beta-band spectral power. Fig. 7A and B show the linear regression for 13 patients with PD in Domain 1. Fig. 7C and D show the linear regression for 11 patients with PD in Domain 2. Fig. 7A and C show putamen BOLD signal predicts the effects of levodopa medication on the beta-band spectral power after the movement onset. Fig. 7B and D show STN BOLD signal predicts the effects of levodopa medication on the beta-band spectral power after movement onset.

Fig. 7 shows the relationship between fMRI BOLD signals in both the contralateral putamen and STN and the difference in movement-related beta-band desynchronization between PD off medication and PD on medication at each domain (putamen and Domain 1: (F(1, 11) = 7.651, *p* = 0.018; STN and Domain 1: (F(1, 11) = 22.011, *p* = 0.001; putamen and Domain 2: (F(1, 9) = 5.349, *p* = 0.046; STN and Domain 2: (F(1, 9) = 29.980, *p* < 0.001)). Patients with PD with a greater BOLD signal in the putamen and STN off medication had the greatest response to dopaminergic medication in the cortex.

## 4 Discussion

The current study used multimodal non-invasive brain imaging to examine cortical and subcortical task-related activity in the basal ganglia and cortex in healthy controls and patients with PD both on and off dopaminergic medications. We present three novel findings. First, patients with PD had exaggerated movement-related beta-band desynchronization in the SMA shortly after movement onset compared to normal controls that was reduced by levodopa. This reduction in movement-related beta desynchronization by levodopa was associated with an increase in movement velocity and in voltage of the biceps agonist burst. Second, we observed a significant positive relationship between beta-band spectral power during movement and movement velocity. Third, we found that greater fMRI BOLD signal in the putamen and STN in the unmedicated state predicted a greater response of beta-band cortical activity to dopaminergic medication. These findings provide new evidence for the importance of beta-band oscillations in the SMA in PD, with particular emphasis on the beta-band and the relation to movement velocity.

### 4.1 Abnormal ballistic movements and exaggerated SMA movement-related beta-band desynchronization in PD

The SMA contributes to motor preparation, sequence learning and initiation of movement (Goldberg, 1985; Tanji, 1996; Nachev et al., 2008) and is connected to the basal ganglia through the thalamus (Akkal et al., 2007). The loss of substantia nigra dopaminergic neurons associated with PD is known to affect the basal ganglia-thalamo-cortical pathway (Albin et al., 1989; DeLong, 1990; Shepherd, 2013) and in particular the supplementary motor area (SMA). Numerous prior studies have shown that SMA functioning is affected in patients with PD (Jenkins et al., 1992; Playford et al., 1992; Rascol et al., 1992, Rascol et al., 1994; Jahanshahi et al., 1995; Haslinger et al., 2001; Grafton et al., 2006; Heinrichs-Graham et al., 2014b, 2014a). For instance, using positron emission tomography (PET) or functional magnetic resonance imaging (fMRI), patients with PD had less regional cerebral blood flow (rCBF) (Jenkins et al., 1992; Playford et al., 1992; Rascol et al., 1994) or blood oxygen level dependent (BOLD) activity (Haslinger et al., 2001) in the SMA during a hand-joystick motor task.

In the current study, we observed that unmedicated patients with PD have exaggerated movement-related beta-band desynchronization (i.e., an exaggerated movement-related reduction in spectral power in the beta-band starting shortly before movement onset) in the SMA compared to healthy controls, and that levodopa reduced this abnormally excessive beta desynchronization. A key contribution of the current study is that the specific timing of the beta-band desynchronization was found prior to movement onset, and during the initial burst phase of the movement when the limb is accelerating toward the target. In the past, the large majority of studies investigating beta-band sensorimotor cortical oscillations in PD have focused on the *resting state*. Both MEG and EEG studies have shown that patients with PD have reduced resting state beta-band spectral power within the primary motor cortex (M1) and primary somatosensory cortex (S1) compared to healthy controls (Stoffers et al., 2007; Pollok et al., 2012; Heinrichs-Graham et al., 2014a; Stegemöller et al., 2016), but not all studies have found this result (e.g., Pollok et al., 2012) including the current study that focused on SMA.

In studies of *movement-related changes* in cortical beta-band oscillatory activity, most (Brown and Marsden, 1999; Heinrichs-Graham et al., 2014b; Stegemöller et al., 2016) but not all (e.g., Moisello et al., 2015) studies have shown that patients with PD exhibit abnormal movement-related beta-band cortical oscillations compared to healthy controls when measured using either electroencephalography (EEG) or magnetoencephalography (MEG). While most studies have found alterations in movement-related spectral power in the beta-band associated with PD, *the direction and magnitude* of these abnormalities have not been consistent across studies. A survey of these studies shows that variability in modality, technique, and movement paradigm are all likely contributing to the lack of consensus regarding movement-related changes in beta-band spectral power.

For example, in a recent EEG study that involved repetitive finger movements including both flexion and extension back and forth, patients with PD (both PD off medication and PD on medication) had excessive and prolonged movement-related beta-band desynchronization compared to healthy controls at the M1 and S1 electrodes (C3 or C4 from EEG) (Stegemöller et al., 2016). This pattern of desynchronization is consistent with the pattern of findings in the current study. It is important to note that the flexion movements occurred at 1–3 Hz, and are difficult to compare to the present study in which a single cued ballistic movement was performed within each 12-second trial. Further, the event-related spectral analysis was confined to the electrodes directly overlying the contralateral M1, while the current study used a statistically-based source analysis procedure including independent component analysis techniques with probabilistic spatial representation of source locations (MPA). Using a stop signal paradigm, it was found that dopaminergic medication increased beta-band power during the stop phase (frontal cortex increase) and go phase (sensorimotor cortex increase) of the task (George et al., 2013), and this set of findings for the sensorimotor cortex is consistent with our current findings. Similarly, another study (Rowland et al., 2015) recorded intraoperative local field potential and high-resolution (4 mm spacing) sensorimotor electrocorticography (ECoG) recordings during an upper limb computer-controlled reaching task during DBS surgery in patients with PD and essential tremor (healthy controls could not be evaluated for obvious ethical reasons). They found that patients with PD had a greater desynchronization in beta-band activity during early movement preparation compared to patients with essential tremor despite similar resting state beta-band spectral power, which is also in agreement with our findings. The authors interpreted this finding as a potential compensatory mechanism to counteract exaggerated beta synchronization in the basal ganglia and attributed to the variability of previous studies regarding movement-related changes in beta-band synchronization to heterogeneity in task and body part involved (Rowland et al., 2015). Yet this study used a more complicated multi-joint reach to target paradigm and surgically implanted ECoG strips rather than EEG.

Conversely, EEG and MEG studies have also reported a decrease in movement-related desynchronization from baseline. For example, in a prior study of movement-related cortical dynamics in patients with PD (Heinrichs-Graham et al., 2014b), patients with PD were found to have less desynchronization in the beta-band when compared with healthy controls during a finger tapping movement. However, the finger tapping task used in their study included flexion and extension phases of two separate movements occurring within a few hundred milliseconds (shown in their Fig. 2), while the current study focused on the ballistic phase of a single more proximal flexion movement. Further, they used MEG (which is superior for detecting currents tangential to the scalp surface but relatively insensitive to radial source currents in the cortex) rather than EEG (which is sensitive to both radial and tangential sources but has a lower spatial resolution) as was used in this study.

Importantly, the current study found that increasing flexion upper limb ballistic movement velocity was associated with increased movement-related beta-band ERSP in the SMA in patients with PD, and we observed significant correlations both off and on dopaminergic medications. Turner et al. (2003) have shown with a sinusoidal tracking task that the joystick movement velocity was positively related to rCBF in the SMA and basal ganglia when patients were tested off medication. Collectively, the findings by Turner et al. (2003) combined with the current data suggest that the relation between rCBF and beta-band power in the SMA may not be a simple linear relation. An important novel contribution of the current study not addressed using rCBF was that the beta-band ERSP changes observed in the SMA between PD and controls occurred about 100 ms prior to movement onset. Since this timeframe is before proprioceptive feedback could affect the cortical activation patterns, this suggests that the changes in PD related to SMA are in the ballistic phase of the movement rather than in the feedback phase of the movement.

### 4.2 Dopaminergic medication effects on the basal ganglia and cortex pathway in PD

We showed in the current study how basal ganglia fMRI activity from the direct and indirect pathway is related to levodopa-induced changes in neuronal oscillations within the SMA in PD. It is well established that fMRI activity within the basal ganglia nuclei increase during a force generation task, and this activity is reduced in early stage PD compared with healthy adults (Spraker et al., 2007; Prodoehl et al., 2010). In a longitudinal study, it was found that patients with PD have a larger reduction in fMRI activity in the putamen and motor cortex than healthy controls over a 1-year period (Burciu et al., 2016). Here, we found that fMRI activity in the putamen and STN in PD off medication predicted the response to dopaminergic medication in movement-related beta-band ERSP within SMA. Our findings suggest that monitoring fMRI activity within the putamen and STN prior to medication administration might assist in stratifying patients in clinical trials testing potential medications for PD.

Among the current treatment options in patients with PD, levodopa (which is converted into dopamine and replenishes depleted striatal dopamine in the basal ganglia) is the most efficacious medication for controlling the motor symptoms of PD, particularly bradykinesia. Levodopa has been shown to increase movement velocity in patients with PD in both the upper or lower limbs (Baroni et al., 1984; Vaillancourt et al., 2004, Vaillancourt et al., 2006). Brown and Marsden (1999) showed that levodopa rendered the attenuation of beta-band power in the M1 electrode (C3 or C4 from EEG) with increased wrist movement amplitude.

Kühn et al. (2006) using local field potential (LFP) at the resting state revealed that levodopa reduced beta-band percentage power in the STN which was associated with an improvement in UPDRS-III scores for the hemibody bradykinesia and akinesia-rigidity subscore. During simple hand-joystick movements, levodopa increased motor-related fMRI BOLD signal of early PD in the SMA and pre-SMA, and decreased in M1, lateral premotor, and superior parietal cortex (Haslinger et al., 2001). Moreover, fMRI BOLD signal in the putamen and thalamus was increased during bimanual power-grip force movements by acute levodopa administration (Kraft et al., 2009). The key observations here that extend the literature are that the task-related fMRI activity within the putamen and STN are associated with the degree to which cortical neuronal activity responds to dopaminergic medication during a ballistic upper limb flexion movement. Given that putamen provides inhibitory input to the direct and indirect pathway, it could be that our results are confined to the indirect pathway, or include the direct and indirect pathway. These new data provide insight into the individual differences that exist across patients with PD in response to dopaminergic medication.

### 4.3 Are beta-band oscillations anti-kinetic?

The findings in this study raise the question are beta-band oscillations in the cortex anti-kinetic? There is little debate that at rest, the LFP recordings in the STN have increased power in patients with PD that relates to clinical symptoms of PD (Kühn et al., 2006). This finding, coupled with the decrease in beta-band power in the motor cortex during a movement has been interpreted to suggest that beta-band oscillations are anti-kinetic (Jenkinson and Brown, 2011). Our findings are in agreement with this interpretation, but also raise some interesting observations. During the ballistic movement, we observed that the beta-band ERSP decreases during movement for controls and patients with PD, and that the beta-band ERSP decreases even further in PD relative to controls. These findings do not necessarily contradict the interpretation that beta-band oscillations are anti-kinetic, but instead suggest that further decreases in beta-band oscillations can signify that the cortex is not functioning normally. It could be that in the cortex, beta-band oscillations operate as a U-shaped function such that a certain degree of beta-band desynchronization is necessary for movement, but excessive beta-band desynchronization can slow movements down. If this is the case, one would expect to observe a positive relation between movement velocity and beta-band ERSP and that levodopa would increase beta-band ERSP, as found in the current study for patients with PD. The importance of the current findings for understanding the pathophysiology of PD is they point to a model where reducing beta-band oscillations are necessary for movement to occur, but that abnormally low beta-band power in the cortex during the ballistic phase of a movement associates with slow movements.

### 4.4 Limitations

There were several limitations to this study. We focused on the acute dopaminergic medication effects on cortical oscillations and tested patients with idiopathic PD in the early stage who were taking dopaminergic medication chronically. It is possible that 12-hour withdrawal from dopaminergic medication was not enough time to wash-out all medication effects (Sossi et al., 2010). In addition, prior studies have focused on beta oscillations in the rebound period several seconds after the movement, whereas our focus was on the ballistic phase during a movement. It is possible that an analysis focused on a greater duration before or after the movement could find different results. Finally, our study focused on frequency bands between 3 and 50 Hz and it is possible that group or medication effects could occur outside of this bandwidth.

### 4.5 Conclusion

In summary, we observed that an acute dose of dopaminergic medication led to a reduction of the abnormally pronounced movement-related beta-band desynchronization in the SMA observed in patients with PD, and increased both ballistic upper limb movement velocity and voltage of the biceps agonist burst. Beta-band spectral power after movement onset was positively correlated with movement velocity. Moreover, we found that greater fMRI BOLD signal in the putamen and STN assessed off medication was associated with a greater response of movement-related beta-band activity in the SMA to dopaminergic medication. Collectively, these findings help to elucidate how cortical oscillations relate to movement velocity during the ballistic phase of movement in PD and demonstrate how BOLD activity in the direct and indirect basal ganglia pathway relate to the modulation of movement-related cortical oscillations by dopaminergic medications.

## Conflict of interest

The authors declare no competing financial interests.
